# GSAn: an alternative to enrichment analysis for annotating gene sets

**DOI:** 10.1093/nargab/lqaa017

**Published:** 2020-03-14

**Authors:** Aaron Ayllon-Benitez, Romain Bourqui, Patricia Thébault, Fleur Mougin

**Affiliations:** 1 University of Bordeaux, Inserm UMR 1219, Bordeaux Population Health Research Center, team ERIAS, Bordeaux 33000, France; 2 University of Bordeaux, CNRS UMR 5800, LaBRI, Bordeaux 33400, France

## Abstract

The revolution in new sequencing technologies is greatly leading to new understandings of the relations between genotype and phenotype. To interpret and analyze data that are grouped according to a phenotype of interest, methods based on statistical enrichment became a standard in biology. However, these methods synthesize the biological information by *a priori* selecting the over-represented terms and may suffer from focusing on the most studied genes that represent a limited coverage of annotated genes within a gene set. Semantic similarity measures have shown great results within the pairwise gene comparison by making advantage of the underlying structure of the Gene Ontology. We developed GSAn, a novel gene set annotation method that uses semantic similarity measures to synthesize *a priori* Gene Ontology annotation terms. The originality of our approach is to identify the best compromise between the number of retained annotation terms that has to be drastically reduced and the number of related genes that has to be as large as possible. Moreover, GSAn offers interactive visualization facilities dedicated to the multi-scale analysis of gene set annotations. Compared to enrichment analysis tools, GSAn has shown excellent results in terms of maximizing the gene coverage while minimizing the number of terms.

## INTRODUCTION

Over the past decade, the revolution in new sequencing technologies has strongly supported the production of omics data to improve our understanding of the relations between genotype and phenotype. This research field involves analyzing gene sets to identify their biological function and to synthesize the key annotation information with the objective to help biologists in their interpretation. In this frame, many tools have been developed to support gene set analysis and visualization of annotations. Most of these tools are based on statistical enrichment methods that usually involve two stages: (i) an *a priori* stage that aims to synthesize the annotation by selecting the over-represented terms, and (ii) an *a posteriori* stage to remove the potentially redundant information by using the Gene Ontology (GO) (1) relations. Examples of such enrichment-based tools are g:Profiler (2), clusterProfiler (3) and WebGestalt (4). In g:Profiler, statistically enriched terms are grouped if they share one or more common parent terms. Two filters, named *moderate* and *strong*, then make use of the hierarchical structure of the used ontologies. The specific functionality *simplify* in clusterProfiler provides a score to retain only the most statistically relevant enriched GO terms (obtained by the *EnrichGO* tool) according to semantic similarity measures. WebGestalt does not propose any step to reduce redundancy within enrichment results, but an annotation file free from redundancy may be used as input of the analysis. Other tools like DAVID (5) propose an *a posteriori* stage that clusters the annotation terms that may be related to each other according to the genes they annotate. This stage does not reduce the number of terms but rather categorizes terms according to their use. The results are thus given as lists of related terms and an additional manual expertise is still required to synthesize the information. Moreover, significant limitations of enrichment-based methods have recently been reported (6,7). First, these methods tend to focus on the most studied genes and provide gene set annotation results that may cover a limited number of annotated genes (6,7,8). Moreover, visualization facilities often suffer from a lack of capacity to perform multi-scale analyses which may help users while interpreting their results.

Another solution can take advantage of the ontology structure where terms are hierarchically structured according to the level of detail of information they provide. These approaches, designated as gene functional similarity, are based on semantic similarity methods and aim to compare two genes according to their annotation terms. Literature has given a variety of tools dedicated to the gene-to-gene analysis in order to group the genes sharing similar annotations and includes G-SESAME (9), GFSAT (10), GOGO (11) and GOSemSim (12), which differ, among other things, by the strategy they adopt for calculating similarity. These approaches have the advantage to compute a comparison score between terms and the extension of such computation to deal with a gene set (that may contain a large number of genes) is certainly of great interest to synthesize the gene set functional information.

Herein, we developed a novel gene set annotation web server, called *Gene Set Annotation* (GSAn). The implemented method uses semantic similarity measures that allow users to *a priori* reduce a large number of GO terms by computing a synthetic annotation for a given gene set. The originality of this new approach is to identify the best compromise between the number of retained annotation terms that has to be drastically reduced and the number of related genes that has to be as large as possible. Moreover, GSAn provides interactive visualization facilities dedicated to the multi-scale analysis of gene set annotations. The GSAn website is available at: https://gsan.labri.fr, the source code is licensed under the MIT licence (https://github.com/Ayllonbe/gsan/tree/Release_1.0.1) and is also available from the release repository at: https://zenodo.org/record/3602010.

## MATERIALS AND METHODS

**Figure 1.**
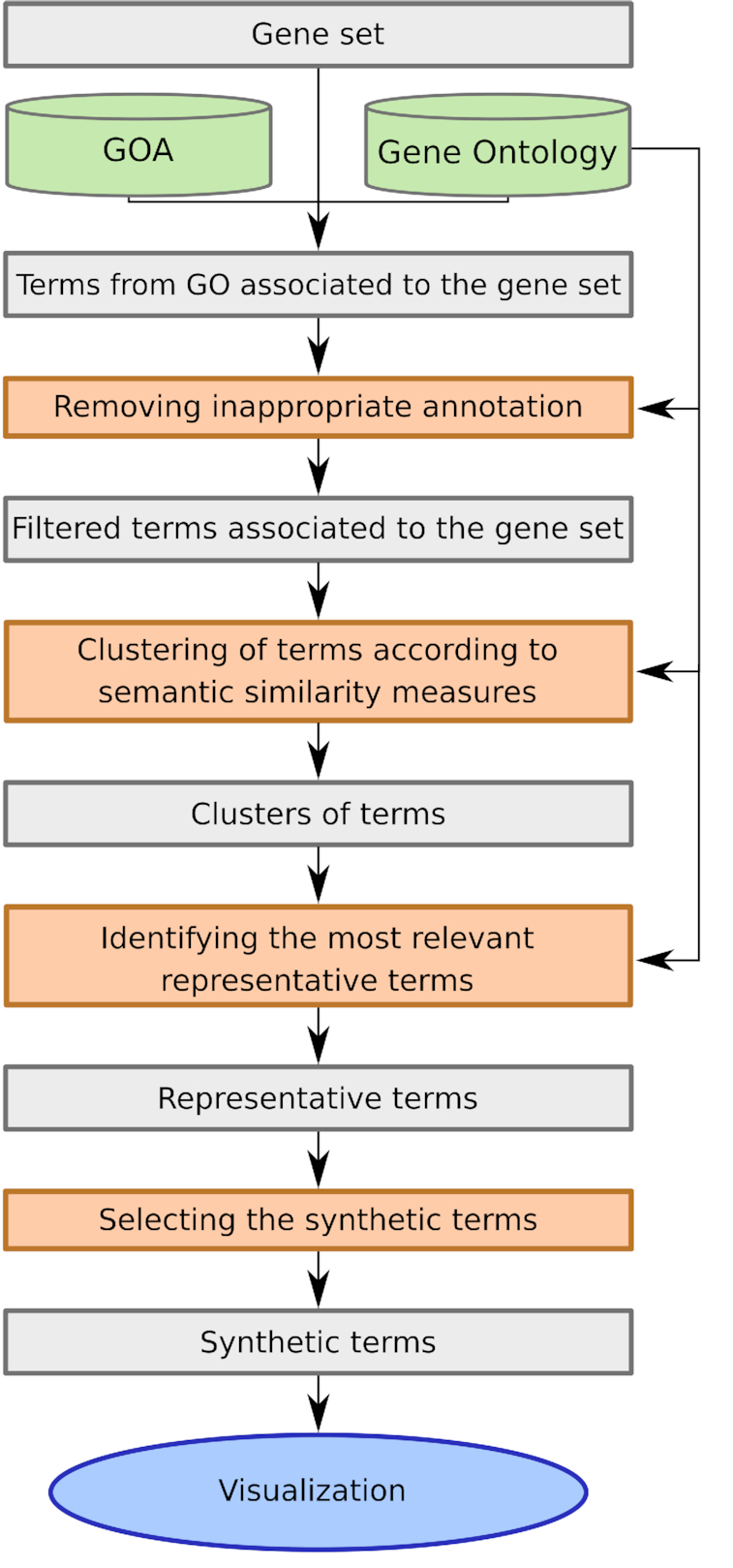
The GSAn method workflow. Steps are represented in dark grey (orange in the electronic version) rectangles while their input and output are displayed as gray rectangles.

GSAn, dedicated to the gene set annotation, is based on a method that makes use of the annotations from Gene Ontology Annotation (GOA) (13) and the hierarchical structure of GO. The method is composed of four main steps which are described in the following paragraphs and summarized in Figure 1. The general workflow is based on findings of a previous study which aimed at studying the impact of using a semantic similarity measure over another one for annotating a gene set (14). All steps, except for step 2, have been improved within GSAn (details about the differences between the GSAn method and the method used in (14) are provided in Supplementary Figure S1).

### Removing inappropriate annotation

The first stage aimed at removing inappropriate annotations, being GO terms that do not provide relevant information. An annotation can be inappropriate for two reasons: redundancy and incompleteness.

Two features can help to identify redundancy: associated evidence and term relationships. For the first feature, GOA provides an evidence code for each gene-term association that explains how this annotation was acquired. Additional information such as *NOT*, *contributes_to* and *colocalizes_with* are specified for qualifying some annotations. Thus, the annotations that were exclusively associated with the evidence code *ND* (no biological data available) or with the qualifier *NOT* were removed. As for the second feature, redundancy corresponds to situations where a gene is annotated by two GO terms hierarchically related. In such case, only the association involving the most specific GO term was retained. Moreover, considering the regulatory relationships within GO, we assumed that a gene associated with a term that regulates another term is also involved in the regulated term. Thus, all regulation terms have been replaced by their regulated terms. For example, the term *regulation of ion transport* (GO:0043269) was replaced by *ion transport* (GO:0006811).

The notion of incomplete annotation was first reported by Faria *et al*. (15) that considered terms with more than 10 descendants as inappropriate (being too general). We adapted this definition of incomplete annotation for taking into account the quality of the annotation. To this end, we considered the information content (IC) defined by Mazandu and Mulder (16) as follows:

(1) \begin{equation*} IC(t) = -log(p(t)) \end{equation*}

where the probability *p*(*t*) is computed considering the position of a term *t* within the hierarchy of a given ontology, as follows:

(2) \begin{equation*} p(t)= \left\lbrace \begin{array}{@{}l@{\quad }l@{}} 1 & \text{if}\ t\ \text{is the root}.\\ \displaystyle \prod _{t_i \in Anc(t)} \frac{p(t_i)}{|Desc(t_i)|} & \text{otherwise}. \end{array}\right. \end{equation*}

where *Anc*(*t*) and *Desc*(*t*) correspond to the list of ancestor and descendant terms of *t* in the hierarchy, respectively.

We computed the IC distribution of terms from GO and we retained only GO terms whose IC was higher than the value of the first quartile.

### Clustering of terms according to semantic similarity measures

Semantic similarity compares GO terms depending on ontological or annotation features. A pairwise semantic similarity measure is defined as a function that, given two terms, returns a value reflecting how close in meaning they are (17,18). A semantic similarity matrix was thus computed for each pair of GO terms associated with the gene set. The semantic similarity measures implemented in GSAn are: Resnik (19) normalized according to Jain and Bader’s approach (20), Lin (21), Aggregate Information Content (AIC) (22), NUnivers (16) and Distance Function (23). Formulas of these semantic similarity measures are available in Supplementary Subsection 2.2. This matrix was then used to compute groups of terms according to the average linkage clustering algorithm (that exhibited the highest cophenetic correlation compared with other algorithms considered in (14)). The best number of clusters was determined using the Average Silhouette Width score (24).

### Selecting the most relevant representative terms

We defined a *representative term* as a term that exhaustively represents the various information given by the terms of a cluster. As the number of representative terms may vary according to the size of the cluster, two strategies were used to determine the best number. First, if a single term inside a cluster annotated more than 70% of genes, it was directly considered as representative. Second, if such a term did not exist, the MSRT algorithm described in (14) was applied to compute an appropriate number of representative terms for the cluster.

For each cluster of terms, the MSRT algorithm aims to find the best compromise between a few number of terms (that has to be small) and a highly specific biological meaning. Starting from the root of the ontology (i.e. *biological process*), the objective was to identify a subset of descendant terms (thus being more specific in their biological meaning) that covers the same set of genes annotated by the terms within the cluster. If multiple subsets of representative terms were obtained, only the one having the best IC mean value was retained.

At the end of this stage that has been applied to each cluster, a new set of terms is obtained from the addition of representative terms of each cluster.

Then, to retain the most relevant representative terms, we used two quality criteria: term redundancy and gene coverage.

#### Removing inappropriate representative terms

Some clusters of terms may have been generated from terms with low similarity between them according the cutting threshold computing by the Average Silhouette Width, resulting in very general representative terms. We thus removed terms whose IC is lower than the first quartile. A new selection stage was then applied to eliminate potential redundancies. According to the type of hierarchical relationship (*is_a* or *part_of*), the removal of the ancestor terms may have a different impact on the number of annotated genes. To deal with this issue, a different strategy was applied according to the type of hierarchical relationships. For the *is_a* relationship, the representative terms being ancestors of other representative terms were removed. For the *part_of* relationship, only the parent or child terms annotating the largest number of genes were retained.

#### Filtering representative terms according to the gene coverage

To filter out the representative terms associated with a limited number of genes, we used a formula that depends on the size of the gene set provided as input. The resulting filtering value This filtering value computes the minimal number of genes for a given gene set and it gradually increases according to the number of genes. For a given gene set, the number of genes of this set is used to determine the minimum number of genes that must be annotated by each representative term. This threshold increases by steps based on the size of the gene set according to the following formula:

(3) \begin{equation*} f(gs)={\rm floor}(\sqrt{|\frac{Ngs}{10}-1|})+2 \end{equation*}

where *gs* is the gene set and *Ngs* is the number of genes in *gs*.

### Selecting the synthetic terms

At last, a heuristic algorithm based on the set cover problem (SCP) (25) was applied to the representative terms for selecting the terms that best summarize the biological information within the gene set, hereafter called *synthetic terms*. In this framework, we thus defined a solution of the SCP as a minimal set of terms covering the maximum number of genes of the gene set.

For a set *R* of representative terms and a gene set *G* whose genes are now annotated by at least a representative term, the synthetic terms were identified according to an iterative process. At each iteration, a score was computed for each representative term and the representative term with the biggest score was then added to a set *S* that gathers synthetic terms. This score is based on the number of genes annotated by a given representative term that are not yet covered by terms within *S* and on a weighted score associated with each representative term. This weighted score takes into account the IC of a term and the number of genes it annotates and is computed as follows:

(4) \begin{equation*} w(t) = \frac{-{\rm log}(\frac{{\rm annotated\_genes\_in\_genome}(t)}{{\rm nb\_genes\_in\_genome}})}{-{\rm log}(\frac{{\rm annotated\_genes\_in\_set}(t)}{{\rm nb\_genes\_in\_set}})} \end{equation*}

where ${\rm annotated\_genes\_in\_set}(t)$ (respectively ${\rm annotated\_genes\_in\_genome}(t)$) corresponds to the number of genes annotated by the term *t* in the gene set under investigation (respectively within the whole) and ${\rm nb\_genes\_in\_set}$ (respectively ${\rm nb\_genes\_in\_genome}$) is the total number of annotated genes within the gene set (respectively within the whole genome). In this formula, a relative measure (expressed as a ratio) has been used to evaluate the quantitative relation between two amounts of terms. The numerator actually corresponds to the IC proposed by Resnik (19). The pseudo-code of the synthetic algorithm (*SA*) and the customized implementation of the SCP are described in Supplementary Subsection 1.2.

### GSAn input

At first, users have to upload a gene or gene product list and to select the appropriate organism within the form. Fourteen organisms are currently stored in GSAn, downloaded from the GO website (http://www.geneontology.org/page/downloads) and from the European Bioinformatics Institute website (https://www.ebi.ac.uk/GOA/downloads), and listed in Supplementary Subsection 2.1. To be more flexible, users can also upload the annotation of any organism of interest using the *GAF* 2.1 format (http://www.geneontology.org/page/go-annotation-file-gaf-format-21). Users may choose any of the three GO sub-ontologies (being biological process or BP, molecular function or MF and cellular component or CC) or any combination of them. If more than one sub-ontology is chosen, the analyses are computed separately and results are then merged. Five semantic similarity measures are available (see the list in ‘Materials and Methods’ section). By default, GO annotations inferred automatically (evidence code: *IEA*) are included in the analysis but users may decide to exclude such annotations.

To customize the analysis, two advanced parameters are proposed to users: the gene support and the incomplete information filter. The gene support is the minimum number of genes that have to be associated to each representative term. The default value of this parameter is determined according to Formula (1) (based on the size of the gene set) and can be modified. The incomplete information filter is used to discard terms presenting a low specificity in the ontology. Four levels of tolerance (none, low, medium and hard) can be applied, corresponding to the percentile values given by the IC distribution (1, 10, 25 and 50, respectively) of GO terms. As a result, terms below the chosen percentile value are discarded. Optionally, users can provide their email address to be notified when the analysis is finished.

The input parameters to be used in GSAn for the analysis are listed in Table 1.

**Table 1.** Input parameters to be used in GSAn for the analysis

Parameter	Description	Default value
Gene list	A list of gene identifiers.	
Genome annotation	Organism name that will be used to recover the gene annotations. Fourteen organisms are proposed and any other organism can be uploaded using the GAF 2.1 format.	homo_sapiens
Inferred from electronic annotation (IEA)	Boolean that indicates whether the IEA annotations should be used.	true
Ontology	Sub-ontology of GO (BP, MF, CC) to be used for the analysis.	BP
Semantic similarity measure	Measure to be used for computing the term similarity matrix.	AIC
**Advanced parameter**		
Gene support	Minimal number of genes that have to be covered by any representative term.	see Formula 3
Incomplete information filter	Tolerance degree to discard terms with a low specificity.	medium
Email	Provided by users for being notified when the analysis is finished.	

### GSAn output

**Figure 2.**
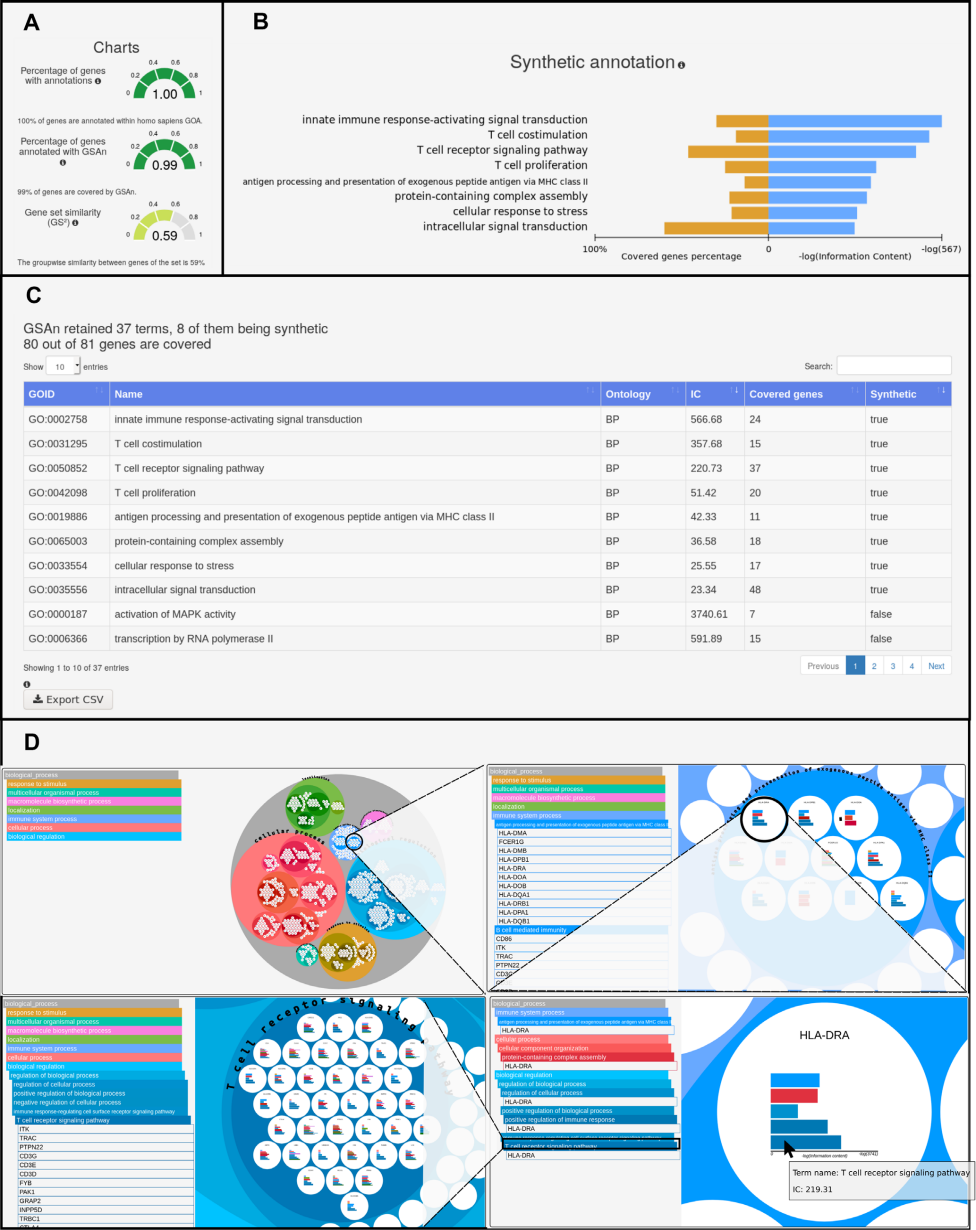
GSAn output results. (**A**) Three gauge plots show information about the annotated genes and the genes covered by GSAn as well as the groupwise similarity of genes in the set defined in (26). (**B**) A diverging bar plot displays the IC and the gene coverage of each synthetic term. (**C**) A table presents all information about representative terms. (**D**) An example of the combined visualization shows the click and zoom interactions.

GSAn results are presented according to multiple visual metaphors. At the top left, three gauge plots display the global gene set information (see Figure 2A). The first one indicates the percentage of genes which are annotated by GO terms while the second one provides the percentage of genes part of GSAn results. At last, the gene set similarity consists in a groupwise approach using the gene annotation proposed in (26). A gene set similarity score of 1.0 means that all genes in the set have the same annotation and 0.0 means that terms have no common annotation. At the top right, a diverging bar plot displays the gene coverage and the IC score of each synthetic term (see Figure 2B). Information about the representative terms is available within two separate pages in two different ways: a table (Figure 2C) and a combined tree visualization (Figure 2D). The table summarizes the information of each representative term, being synthetic or not. The tree visualization aims to describe the hierarchical context of each representative term of the gene set within GO. This tree visualization has previously been described in details in (27) for the annotation of multiple gene sets (each leaf of the tree corresponds to a gene set) and has been adapted for being integrated within GSAn to focus on the gene information (each leaf of the tree corresponds to a gene). To obtain such a visualization, the GO structure (represented as a directed acyclic graph) has been transformed into a tree according to the most informative parent of each representative term. Two types of tree visualizations are then combined: a collapsible indented tree and a circular treemap. A tree color algorithm is applied to attribute similar colors to terms that are hierarchically related (28). The brightness of the circle is related to the depth of terms in the ontology (darker means deeper in GO). White color forms represent the genes inside their annotation terms. Thus, a given gene can appear inside several terms of different branches. Moreover, within each gene circle, a bar chart is displayed to represent its annotation terms (using their assigned colors). This visualization allows to explore annotation results thanks to interactions such as zooming within the circular treemap, or expanding the branch in the indented tree (Figure 2D). Additionally, users can download a JSON file and explore these results again by uploading the file within the ‘Visualization’ page. Also, results can be downloaded as a CSV format.

### Implementation and storing results

GSAn has been implemented in JAVA EE using the SpringBoot framework. From the client side, the page exhibiting results has been implemented based on JavaScript using the D3.js (29) and TreeColors.js (https://github.com/e-/TreeColors.js/) libraries. The releases of GO and GOA are weekly updated. The JSON files created by GSAn are stored during 12 h.

## RESULTS AND DISCUSSION

To illustrate GSAn, we present two analyses: (i) a comparison of the results with known enrichment tools, (ii) an application of GSAn using a gene set involved in the immune system and (iii) an application of GSAn to genes involved in pathways.

For the first two analyses, we used the dataset computed by Li *et al*. (30), called BTM for blood transcriptional modules. This dataset is a repertoire of 346 gene sets characterizing innate and adaptative immune response in vaccination studies and was built using a large-scale data integration of human blood transcriptome provided by the NCBI Gene Expression Omnibus. Moreover, ‘interactome’, ‘bibliome’ and pathways extracted from public databases were integrated to create a set of transcription modules.

For the last analysis, details are provided in Supplementary Section 5. More precisely, seven gene sets have been investigated, for which we present the annotation terms corresponding to the name of the pathway given by each studied tool (see Supplementary Table S1).

### Comparing GSAn to enrichment tools

Due to the fact that GSAn provides an annotation for a given gene set, it has been compared to the following classical enrichment analysis tools: g:Profiler (2), clusterProfiler (3), WebGestalt (4) and DAVID (5) (Table 2). As mentioned in the introduction, each tool provides a stage to reduce the number of terms by eliminating the redundancy, except for DAVID. This comparison investigates the impact of the reduction step to decreasing the number of annotation terms while maintaining the number of annotated genes.

**Table 2.** Comparison of gene set functional analysis tools

	GSAn	DAVID	g:Profiler	clusterProfiler	WebGestalt
**Statistical method**	-	Fisher’s Exact	Hypergeometric	Hypergeometric	Hypergeometric
**Genes id**	Symbol	Many	Many	Many	Many
**Accessibility**	WebSite, Rest Api	WebSite, Rest Api, R	WebSite, Rest Api, R	R	WebSite, R
**Number of organisms**	14*	hundreds	hundreds	2	12*
**Updates**	daily				
**Other resources**	No	Yes	Yes	Yes	Yes
**Reduction step**	Synthetic algorithm, see ‘Materials and Methods’ section for more details		Hierarchical filtering of the terms based on the *P*-value	Score filtering of the terms based on semantic similarity measures	Hierarchical filtering of the terms in the annotation file before computing the analysis

* Users may contact authors if they need an additional organism to be supported within GSAn.

#### Qualitative comparison based on expert assessment

Two aspects were developed in this qualitative comparison using the same data set composed of a group of 84 genes extracted from Li *et al*. (30) (the list of UNIPROT IDs is available in Supplementary Subsection 3.1). First, the end-user experiences was quantified and qualitatively analyzed through a heuristic evaluation with eight experts and six master students. Second, a more detailed analysis was conducted using the tools results from the quality point of view. Based on the recommendations given by Pesquita *et al*. (31), we conducted a domain expert assessment of the tools described in the previous section (except for clusterProfiler which does not offer a web interface).

**Figure 3.**
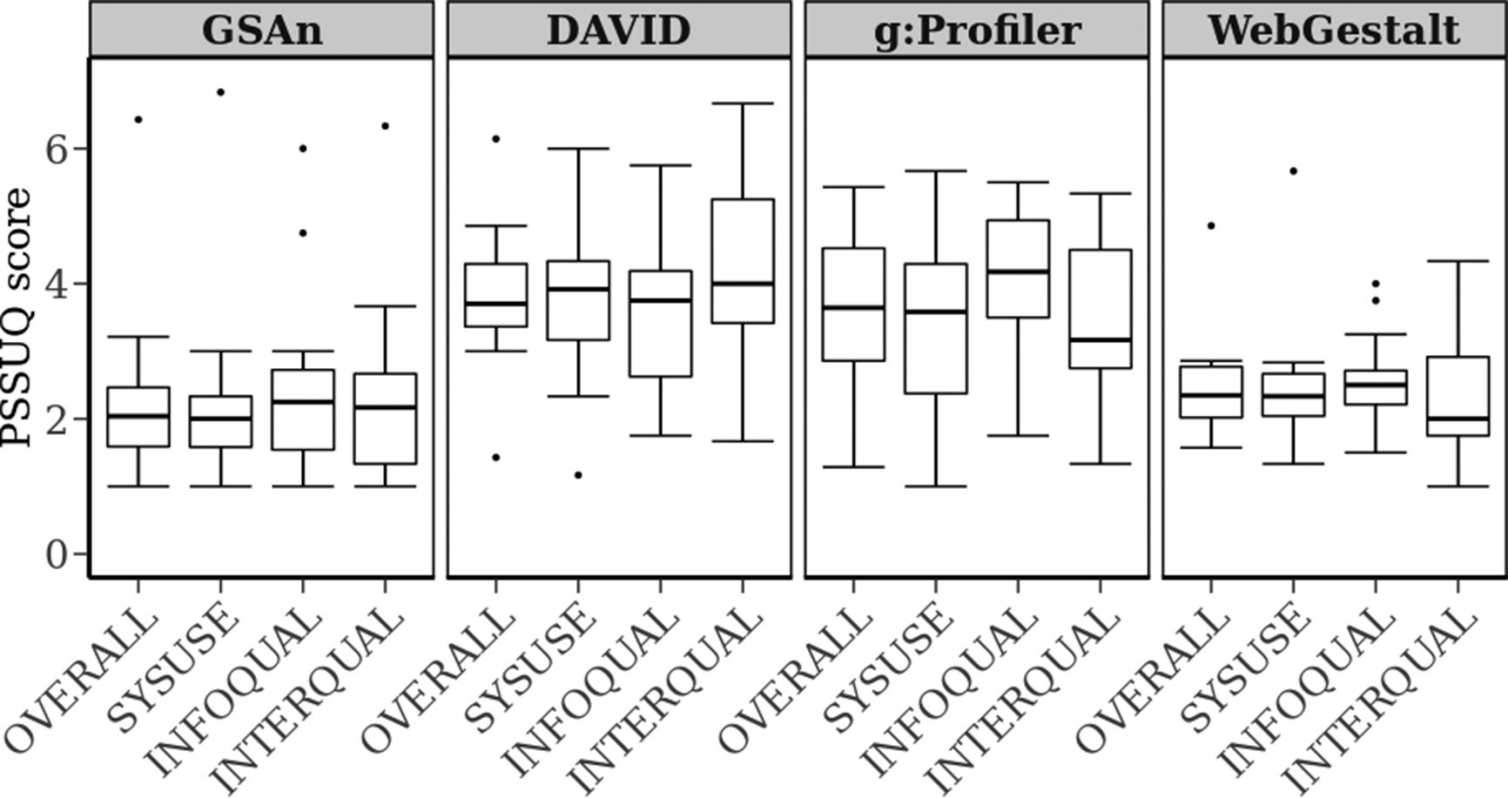
Results of the qualitative assessment. Each boxplot shows the experience of eight experts and seven master students through the PSSUQ questionnaire.

The *purpose* of this investigation was to evaluate the usability of the different tools. The *setup* of this evaluation was based on the Post-Study System Usability Questionnaire (PSSUQ (32)) composed of 16 questions with a seven point Likert scale to evaluate the agreement, ranging from 1 (strongly agree) to 7 (strongly disagree). These items can be analyzed in more details regarding the three sub-scores that reflect different aspects of the tool usability: usefulness (SYSUSE score: six questions), information quality (INFOQUAL score: five questions) and interface quality (INTERQUAL score: four questions). A score has been computed for each of these categories for each evaluated tool. In this scope, we built a *panel of users* composed of domain experts with five medical/pharmacist researchers and three bioinformaticians. In addition to this panel, seven master students with a background in biology performed this evaluation. Both evaluation results are shown in Supplementary Figure S2. Using the tools, the participants had to analyze a set of 84 genes that was constituted by Li *et al*. (30) while analyzing five human vaccines. Participants of the evaluation were asked to process the following *tasks*: (i) Can they identify the function(s) (a maximum of three functions was expected for interpreting the 84 genes) that describe the gene set of interest, (ii) looking at the annotation terms that were proposed, can the user estimate the ratio of the genes that are related to this annotation? The resulting scores displayed in Figure 3 showed that GSAn and WebGestalt both exhibit a different profile from DAVID and g:Profiler. For the two first tools, evaluators provided positive comments regarding the overall score and the three sub-scores and they generally performed the required tasks without difficulty. For the two other tools, median score values were close to the neutral threshold (that corresponds to the score value of 4) with, in addition, a wider score dispersion that demonstrates more nuanced responses by each end-user. The results given by WebGestalt and GSAn follow the same trend with a slightly better evaluation concerning the INTERQUAL score for WebGestalt, closely followed by GSAn.

To go further, a more detailed analysis of the five top terms according to the specific quality criteria for each tool (used as default mode) was conducted. The top five terms provided by each tool were positioned along the GO hierarchy represented on an image constructed using QuickGO (33) (provided as Supplementary Figure S3). The depth corresponding to the top five terms for each tool is ranged from 6 to 10 for GSAn, 2 to 10 for DAVID, 3 to 7 for g:Profiler and 3 to 5 for WebGestalt. Their biological pertinence can be related to their depth within the tree varying from 0 (corresponding to Biological Process) to 15. Most specific terms are proposed by GSAn, with the highest specificity for 3 out of 5 terms. DAVID also proposes very specific terms while giving general terms (two out of five terms with a depth of two or three). The terms given by the other tools have more intermediate levels of specificity. Then, while ascending the tree, by starting with the most specific terms, the number of genes related to each term was computed for each tool and displayed in Table 3. g:Profiler and WebGestalt show a high level of coverage from the first term while the results for DAVID and GSAn give a growth in this coverage, which can be interpreted as a best solution for finding a compromise between a good quality of non redundant information and a high level of biological pertinence.

**Table 3.** Comparison of the top five GO terms provided by each gene set functional annotation tool

Tool	Term	Term ID	Related genes	Cumulative related genes	Tools that also use the term
**GSAn**	Innate immune response-activating signal transduction	GO:0002758	23	23	
	T cell receptor signaling pathway	GO:0050852	37	55	**DAVID**
	T cell costimulation	GO:0031295	15	58	**DAVID**
	Cytokine biosynthetic process	GO:0042089	15	60	
	T cell proliferation	GO:0042098	19	63	
**DAVID**	T cell receptor signaling pathway	GO:0050852	34	34	**GSAn**
	B cell receptor signaling pathway	GO:0050853	14	44	
	T cell costimulation	GO:0031295	26	49	**GSAn**
	Adaptive immune response	GO:0002250	21	53	**WebGestalt**
	Immune response	GO:0006955	26	63	
**g:Profiler**	Immune response-activating signal transduction	GO:0002757	60	60	
	immune response-regulating signaling pathway	GO:0002764	67	67	**WebGestalt**
	Positive regulation of immune system process	GO:0002684	68	70	
	Regulation of immune response	GO:0050776	71	71	
	Regulation of immune system process	GO:0002682	73	73	
**WebGestalt**	Immune response-regulating signaling pathway	GO:0002764	68	68	**g:Profiler**
	Regulation of leukocyte activation	GO:0002694	38	72	
	T cell activation	GO:0042110	35	72	
	Leukocyte cell-cell adhesion	GO:0007159	27	72	
	Adaptive immune response	GO:0002250	34	72	**DAVID**

#### Quantitative comparison using 226 gene sets

Supplementary Figure S4 shows the percentage of genes annotated by GSAn, DAVID, g:Profiler, ClusterProfiler and WebGestalt for the 360 BTM gene sets. To carry out the quantitative comparative analysis, we focused on the GO biological process terms and retained only the BTM gene sets (30) for which annotation was provided by all tools. Two hundred and twenty-six gene sets were thus considered for the analysis. An IC distribution measure was computed based on the IC proposed in (16) and the ontology. This measure is relevant to analyze the gene coverage and the number of terms provided by each tool. We used four thresholds (from Q_0_ to Q_3_) corresponding to the quartiles of the IC distribution to filter the results of each tool. Each threshold filters out the terms with an IC below their value. Thus, Q_0_ refers to the whole resulting GO terms and Q_1_, Q_2_ and Q_3_ correspond to GO terms having an IC value over 18.4, 44.4 and 155.3, respectively.

**Figure 4.**
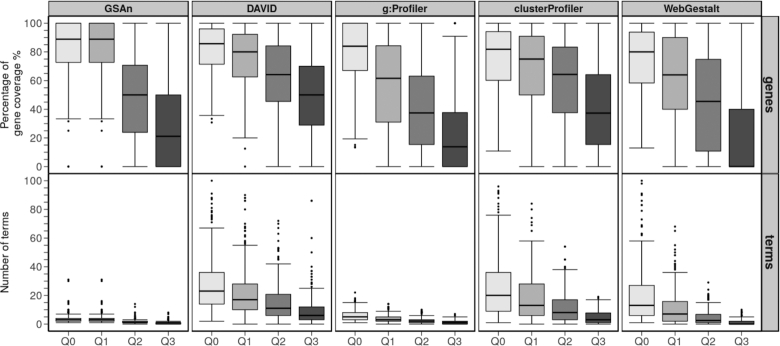
Box-plots providing the impact on the gene coverage percentage and the number of terms for each tool using the BTM gene sets (30) according to the quartile computed by the IC distribution in GO. Each quartile Q_*x*_ corresponds to the IC value according to which the terms are filtered out. Thus, Q_0_ corresponds to the whole set of terms provided by the tools and Q_3_ to terms with an IC value higher than the third quartile of the IC distribution in GO.

Figure 4 displays, for each tool, the gene coverage and the number of terms according to the IC distribution.

Regarding the distribution of the gene coverage in Q_0_, no significant difference has been observed between tools (statistical results are provided in Supplementary Figures S5–12). Thus, all tools present a median value higher than 80% of the complete gene set. On the other hand, regarding the number of terms, two classes of tools can be identified. First, DAVID, clusterProfiler and WebGestalt give a median number of terms ranging from 10 to 25. The second group, involving GSAn and g:Profiler, has a smaller dispersion of numbers according to the various gene set with a median value ranging from 0 to 5. This smaller number of terms combined with a high gene coverage is relevant because very few terms annotate almost the whole genes. Considering Q_1_, all tools except GSAn get decreased gene coverage and number of terms. GSAn keeps the same values as for Q_0_ due to the IC filter applied to remove incomplete information (corresponding to the first quartile in the distribution).

At last, Q_2_ and Q_3_ present the results for the most specific terms. The gene coverage is higher for DAVID and clusterProfiler with a median of gene coverage over 40%. They both lead to a better compromise regarding the number of terms and the gene coverage while maintaining relevant knowledge. However, the high number of terms may suggest that each resulting term is likely to annotate few genes.

**Figure 5.**
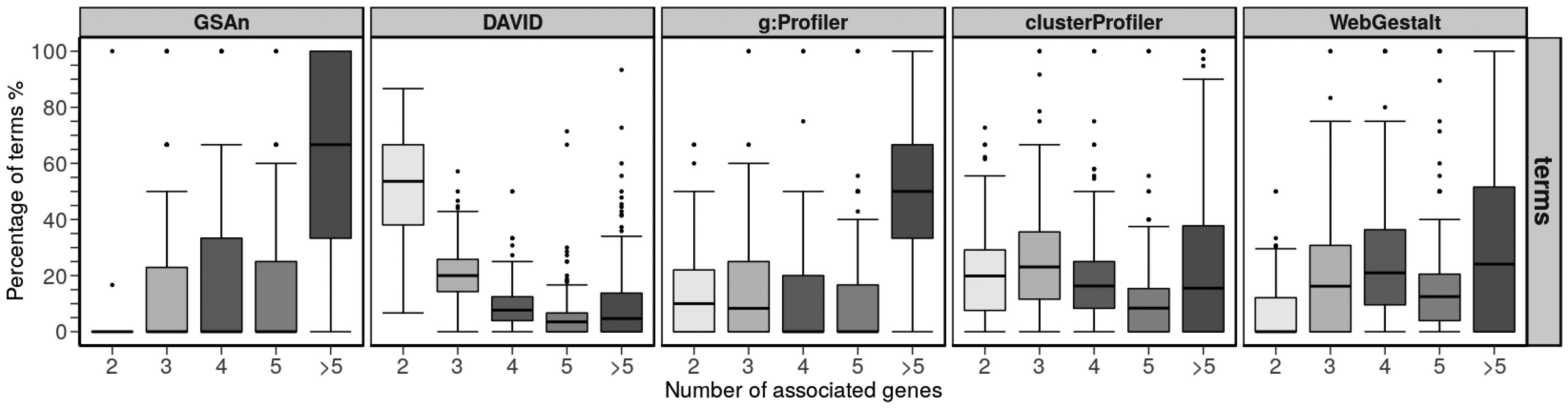
Box-plots showing the classification of terms according to the number of annotated genes. When terms are annotated mainly by 2 or 3 terms, it means that the result presents more specific terms. When terms are annotated mainly by more than 5 terms, it means that the terms are more general but that they are involving more genes.

To further investigate this hypothesis, we analyze the percentage of terms according to the number of genes that are annotated by these terms. Thus, Figure 5 shows the percentage of terms annotating 2, 3, 4, 5 and more than 5 genes. We observe that a median value of 50% of terms provided by DAVID annotate only two genes and the rest of the median boxes does not exceed 20%. On the contrary, the majority of terms provided by GSAn and g:Profiler annotate more than five genes. That suggests the existence of some general terms among GSAn and g:Profiler results, which involve more genes compared to DAVID and clusterProfiler that include terms annotating few genes. Lastly, clusterProfiler and WebGestalt follow a similar behavior, with a median value for each box under 25%.

At last, one should note that the combination of results obtained from GSAn and those of an enrichment tool could be of interest. In such case, the tools have to be used independently because representative/synthetic terms computed by GSAn may not be among the annotations provided by GOA and then not part of the annotations within a whole genome of interest (that are used in enrichment analysis).

### Gene set application by using GSAn

In the second case study, we use GSAn to analyze a BTM module annotated by experts as *regulation of antigen presentation and immune response* (30). This module contains 81 genes involved in the signal transduction in the immunological process against pathogens. The default parameters of GSAn are used and the chosen semantic similarity measure is NUnivers. GSAn retains 37 representative terms covering 80 out of 81 genes and eight of them are synthetic terms (Figure 2). The gauge plots show a high gene coverage using the GOA file (first gauge) and GSAn analysis (second gauge). At last, the third displays a gene set similarity of 0.59, which means that genes share a high number of terms.

By focusing on the synthetic annotation displayed within the diverging bar plot, we observe terms related to the proliferation and co-stimulation of T cells and the activation of signaling transduction by the innate immune response. Also, these terms and the term *antigen processing and presentation of exogenous peptide antigen via MHC class II* (GO:0019886) are consistent with the manual annotation performed by experts and show that the annotation provided by GSAn is even more specific. Indeed, GSAn illustrates that the module is also involved in the proliferation of T cells. Moreover, more complete information may be observed from the representative terms through the information table or the combined tree visualization. By exploring the tree visualization, we obtain additional information, such as terms sharing the same informative ancestor or the genes annotated by more than one term. For example, by focusing on the term *antigen processing and presentation of exogenous peptide antigen via MHC class II*, we notice that eleven genes are annotated by this term. When clicking and developing in details each gene, we observe that six out of the eleven genes are annotated by *T-cell receptor signaling pathway* (GO:0050852) and three of them by *T-cell proliferation* (GO:0042098). Thus, with very few user interactions, we retrieve additional information about the biological role of some genes in the module.

## CONCLUSION

The main problems in finding gene signatures are mainly related to the investigation of the biological function of gene sets. That problem can be solved using classical enrichment methods, such as DAVID or g:Profiler. However, these methods focus on the most studied genes that may provide annotations covering a limited number of annotated genes (6,7,8). Another problem is the redundant information within annotations that may increase the difficulty in interpreting results when no *a posteriori* filter is applied. To address these issues, we propose a new web server as an alternative to classical enrichment analysis. The underlying method makes use of the hierarchical structure of GO to reduce the number of terms while keeping an appropriate level of biological information. Compared to enrichment analysis tools, GSAn has shown excellent results in terms of maximizing the gene coverage while minimizing the number of terms. GSAn has provided a gene set annotation that is more specific than results given by experts (for a human gene set). Also, an originality of GSAn is to provide interactive visualization abilities to analyze the resulting gene set annotations. The underlying visualization is based on a combined tree that supplies zoom operations to browse terms and the genes they annotate according to the level of biological information that may interest users.

## DATA AVAILABILITY

The source code is licensed under the MIT licence (https://github.com/Ayllonbe/gsan/tree/Release_1.0.1) and is also available to reproduce the analyses at: https://zenodo.org/record/3602010.

The generated datasets analyzed in the current study are available in the https://github.com/Ayllonbe/gsan/tree/Release_1.0.1 repository and in Supplementary Data.

## SUPPLEMENTARY DATA

Supplementary Data are available at NARGAB Online.

## Supplementary Material

lqaa017_Supplemental_Files
