# Supplementary material for: GSAn: an alternative to enrichment analysis for annotating gene sets
Source: NAR Genom Bioinform. 2020 Mar 14;2(2):lqaa017. doi: 10.1093/nargab/lqaa017 (PMC7671311; doi:10.1093/nargab/lqaa017)
Supplement: lqaa017_Supplemental_Files [file lqaa017_supplemental_files.zip › ReviewedR2_Ayllon_supplementary_data_nar.pdf]

Supplementary data

Aarón Ayllón-Benítez, Patricia Thébault, Romain Bourqui, Fleur Mougin

Contents

1 Supplementary details on the GSAn method **3**

1.1 Detailed steps of the GSAn method 3

1.2 Pseudocode to identify the synthetic terms 4

2 Supplementary details on GSAn input parameters **5**

2.1 List of organisms available in GSAn 5

2.2 Semantic similarity measures implemented in GSAn 6

2.2.1 Information content 6

2.2.2 Semantic similarity measures 7

3 Supplementary details on the qualitative analysis described in the "Comparing GSAn to enrichment tools" subsection **10**

3.1 List of UNIPROT IDs of the studied gene set 10

3.2 Results of the evaluation made by experts and students 13

3.3 QuickGO view of the top 5 terms provided by each evaluated tool . 14

4 Supplementary details on the quantitative analysis described in the "Comparing GSAn to enrichment tools" subsection 17

4.1 Description of the investigated gene sets 17

4.2 Statistical comparison of results provided by each evaluated tool . . 18

5 Supplementary results by applying GSAn onto real pathway genes 23

1 Supplementary details on the GSAn method

1.1 Detailed steps of the GSAn method

Figure 1 shows the different steps of the GSAn method represented in orange rectangles. The brown dotted ovals correspond to the steps that had not been implemented within the method described in [1].

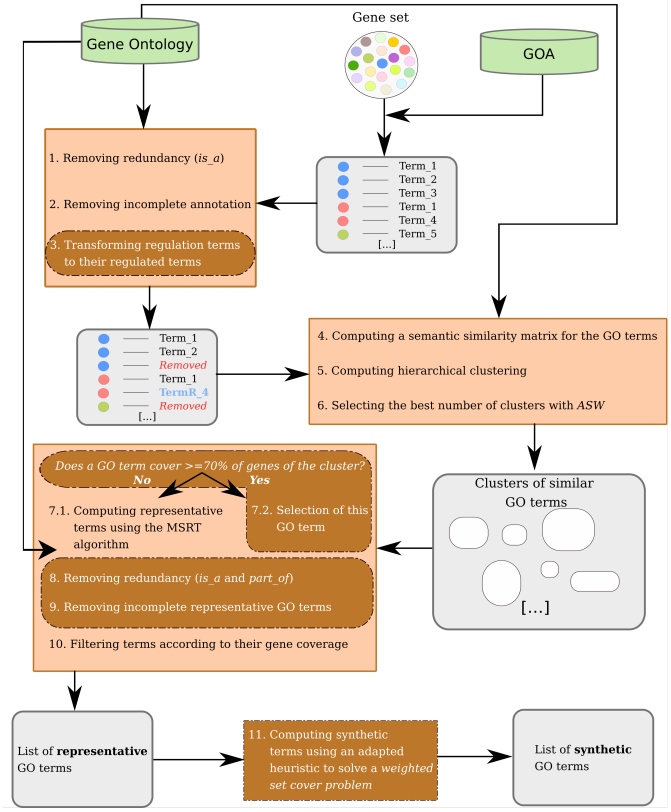

Figure 1: Detailed steps implemented within the GSAn method. Steps are represented in orange rectangles and brown dotted ovals correspond to new steps compared with the method used in [1].

1.2 Pseudocode to identify the synthetic terms

During the last step of GSAn (see the subsection `Selecting the synthetic terms` from the GSAN METHOD section of the main paper), the synthetic terms have to be identified. Thus, to recover them while preserving the same number of covered genes, we applied the following heuristic algorithm that is based on the set cover problem (SCP).

Algorithm 1: $SA(R, G)$

Input : R is a set of representative terms,

G is a set of genes covered by at least one term from R.

Used functions: $genes(x)$ is the list of genes in G annotated by the term x,

$w(x)$ is the weight score of the term x, determined as follows:

$$w(t) = \frac{-log(\frac{annotated\_genes\_in\_genome(t)}{nb\_genes\_in\_genome})}{-log(\frac{annotated\_genes\_in\_set(t)}{nb\_genes\_in\_set})}$$

1) Let S represent the set of synthetic terms and C represent the set of genes covered by all terms in S.

$Initialize\ S := \emptyset$

$Initialize\ C := \emptyset$

2) While C is not the same as G :

a) Find the term $r \in R$ whose score is the biggest, in which score is defined by:

$score(r) := |genes(r) - C| \cdot w(r)$

b) Add the term with the biggest score to S and remove it from R

$S := S \cup \{r\}$

$R := R - \{r\}$

$C := \bigcup_{\forall s \in S} genes(s)$

return S

---

2 Supplementary details on GSAn input parameters

The subsections provide details about the organisms and the semantic similarity measures available in GSAn as well as the way the gene support is calculated.

2.1 List of organisms available in GSAn

Organism	File
Sus scrofa	goa_pig.gaf
Saccharomyces cerevisiae	goa_yeast.gaf
Rattus norvegicus	goa_rat.gaf
Mus musculus	goa_mouse.gaf
Homo sapiens	goa_human.gaf
Gallus gallus	goa_chicken.gaf
Escherichia coli	gene_association.ecocyc
Drosophila melanogaster	goa_fly.gaf
Danio rerio	goa_zebrafish.gaf
Canis lupus	goa_dog.gaf
Candida albicans	gene_association.cgd
Caenorhabditis elegans	goa_worm.gaf
Bos taurus	goa_cow.gaf
Arabidopsis thaliana	goa_arabidopsis.gaf

2.2 Semantic similarity measures implemented in GSAn

Before presenting the semantic similarity measures available in GSAn, we first introduce the notion of information content because it is used in most of these measures.

2.2.1 Information content

The information content, or IC, is a score associated with a term within an ontology, indicating how much this term is informative. The bigger the IC is, the more specific the term is. Two kinds of IC are used in GSAn: *intrinsic* and *extrinsic*. The intrinsic IC uses only information available within the ontology structure and the extrinsic IC uses external information.

Extrinsic IC. The extrinsic IC that is used in GSAn was proposed by Resnik in 1995 [2]. The principle is to compute the frequency of occurrence of a word in a given document. Applied to the Gene Ontology (GO), the frequency of a GO term is determined by the number of genes that are annotated by a GO term within the GOA file of a given organism. Thus, the probability of a GO term is computed as follows:

$$p(t) = \frac{frequency(t)}{frequency(root)} \tag{1}$$

Thus, the extrinsic IC is computed as follows:

$$IC_{ext}(t) = -log(p(t)) \tag{2}$$

This IC is used by some semantic similarity measures, whose formulas are

provided in the next subsection.

Intrinsic IC. The intrinsic IC used in GSAn is the one proposed by Mazandu and Mulder that takes into account the position of GO terms in the GO structure [3]. First, the probability is computed recursively, from the root term to the leaf terms. Each term depends on the probability of its parents divided by the children of the parent. Then, the IC provided by Mazandu for a term t is the following:

$$p(t) = \begin{cases} 1 & \text{if } t \text{ is a } \textit{root}. \\ \prod_{t_p \in parents(t)} \frac{p(t_p)}{|children(t_p)|} & \text{otherwise.} \end{cases} \quad (3)$$

$$IC_{GOu}(t) = -log(p(t)) \quad (4)$$

Hybrid IC. Song *et al.* [4] proposed a hybrid IC combining the semantic value of Wang *et al.* [5] and the extrinsic IC of Resnik [2]. The semantic value and the semantic weight are computed as follows:

$$SW(t) = \frac{1}{1 + e^{-\frac{1}{IC_{ext}(t)}}} \quad (5)$$

$$SV(t) = \sum_{t \in ancestors(t)} SW(t) \quad (6)$$

2.2.2 Semantic similarity measures

Resnik. This semantic similarity measure was proposed by Resnik simultaneously with its IC [2]. The similarity between two terms corresponds to the IC of

their most informative common ancestors (currently designated as $MICA$). The equation, after being normalized according to Jain and Bader's approach [6], is as follows:

$$Sim_{Resnik}(t_a, t_b) = \max_{t_{anc} \in ancestors(t_a) \cap ancestors(t_b)} (IC_{ext}(t_{anc})) \tag{7}$$

where $ancestors(t_x)$ and $IC_{ext}(t_x)$ correspond respectively to the ancestor terms and the extrinsic IC score of the term t_x.

Lin. To improve the results of Resnik's similarity, Lin proposed the following normalization [7].

$$Sim_{Lin}(t_a, t_b) = \frac{2 \cdot Sim_{Resnik}(t_a, t_b)}{IC_{ext}(t_a) + IC_{ext}(t_b)} \tag{8}$$

Aggregate IC (AIC). The aggregate information content or AIC is an alternative measure proposed by Song *et al.* using the hybrid IC [4]. The semantic similarity is thus computed according to the similarity provided by Wang *et al.* [5] as follows:

$$Sim_{AIC}(t_a, t_b) = \frac{\sum\limits_{t_{anc} \in ancestors(t_a) \cap ancestors(t_b)} 2 \cdot SW(t_{anc})}{SV(t_a) + SV(t_b)} \tag{9}$$

NUnivers. Using the IC proposed by Mazandu and Mulder [3], the similarity of NUnivers between two terms is the IC of the most informative content ancestor divided by the maximal IC between the compared terms.

$$Sim_{NUnivers}(t_a, t_b) = \frac{\max\limits_{t_{anc} \in ancestors(t_a) \cap ancestors(t_b)} IC_{GOu}(t_{anc})}{\max \{IC_{GOu}(t_a), IC_{GOu}(t_b)\}} \tag{10}$$

Distance Function. The distance function (DF) is adapted from the Jaccard index [8], as follows:

$$Sim_{DF}(t_a, t_b) = \frac{|ancestors(t_a) \cap ancestors(t_b)|}{|ancestors(t_a) \cup ancestors(t_b)|} \tag{11}$$

3 Supplementary details on the qualitative analysis described in the "Comparing GSAn to enrichment tools" subsection

3.1 List of UNIPROT IDs of the studied gene set

UNIPROT ID list of the data set composed of a group of 84 genes extracted from Li *et al.* [9].

P55160

Q8N1K5

P04440

P05412

P25963

P29350

O60602

P01903

P01730

Q04759

P19174

P20963

P01850

Q92637

P32248

Q9Y2C9

Q9BXR5

O15117

O60603

P30273

Q6PIZ9

Q8N423

Q8NHL6

Q9BXN2

Q7Z6A9

P06241

P09769

P42081

O75791

P01920

P43403

Q9NWQ8

P41218

Q9Y616

P06239

Q16828

Q9Y2R2

Q13153

Q16512

P09668

P16410

Q13094

O00206

P41240

P01100

Q9GZY6

Q9UHI5

Q9BXL7

P01911

O15111

P25063

P57075

P52564

Q9NYK1

Q9Y6Y9

P08631

P50552

P07948

Q9H400

Q16539

Q99836

Q92835

Q08881

P01848

Q9H6Q3

P26718

P28068

Q15418

P01909

Q06413

P43405

Q02078

Q15399

P08575

P06340

P13765

P28067

P20036

P15391

P04234

P07766

Q06187

P09693

3.2 Results of the evaluation made by experts and students

Figure 2 shows the results of the qualitative evaluation performed respectively by experts (panel (A)) and students (panel (B)) when using GSAn, DAVID [10],

g:Profiler [11] and WebGestalt [13].

3.3 QuickGO view of the top 5 terms provided by each evaluated tool

Figure 3 shows the top 5 terms provided by GSAn, DAVID [10], g:Profiler [11] and WebGestalt [13] positioned along the GO hierarchy represented on an image constructed using QuickGO [14].

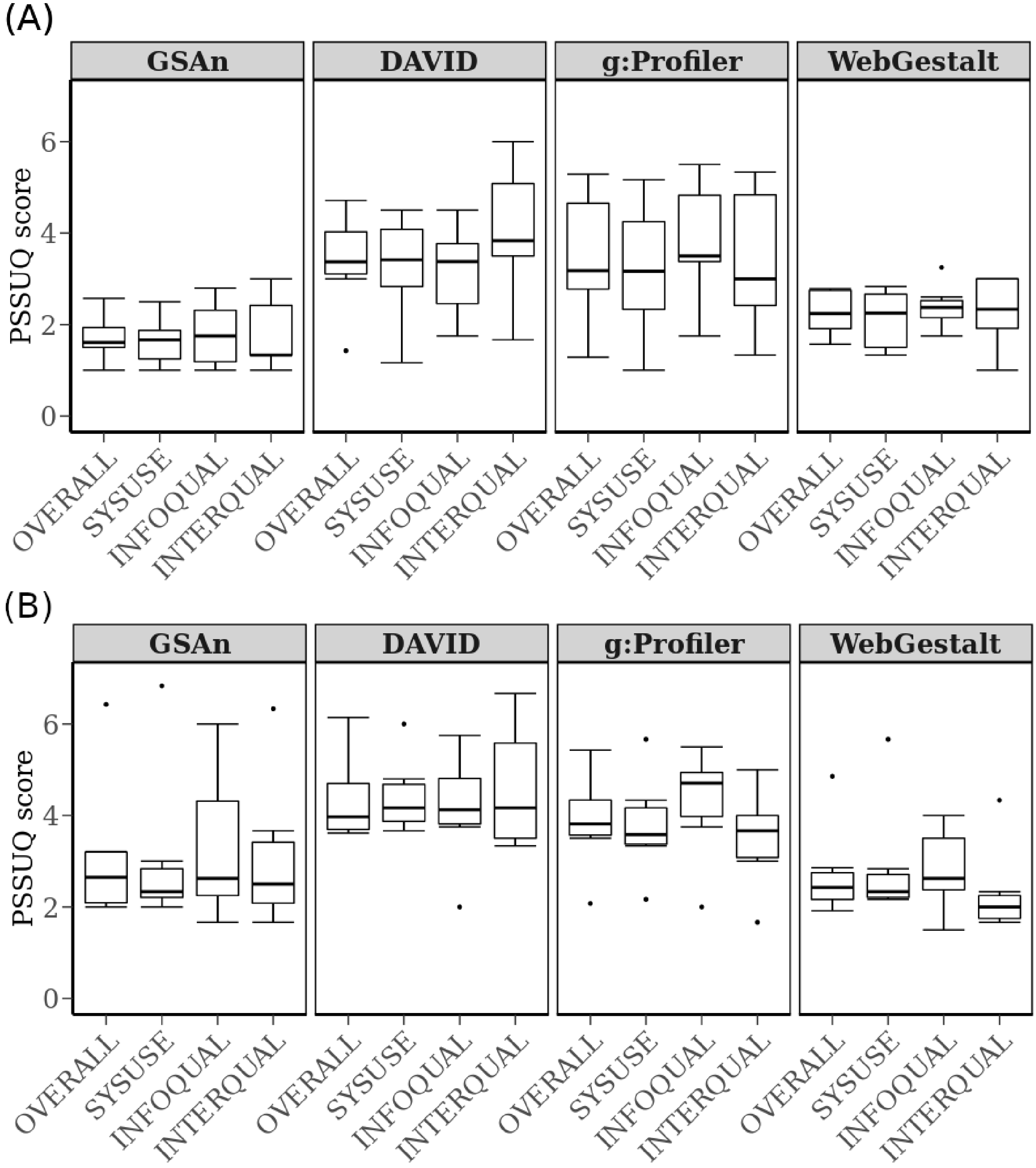

Figure 2: Boxplots showing the experience of evaluators through the PSSUQ questionnaire: (A) assessment of eight experts, (B) assessment of seven master students.

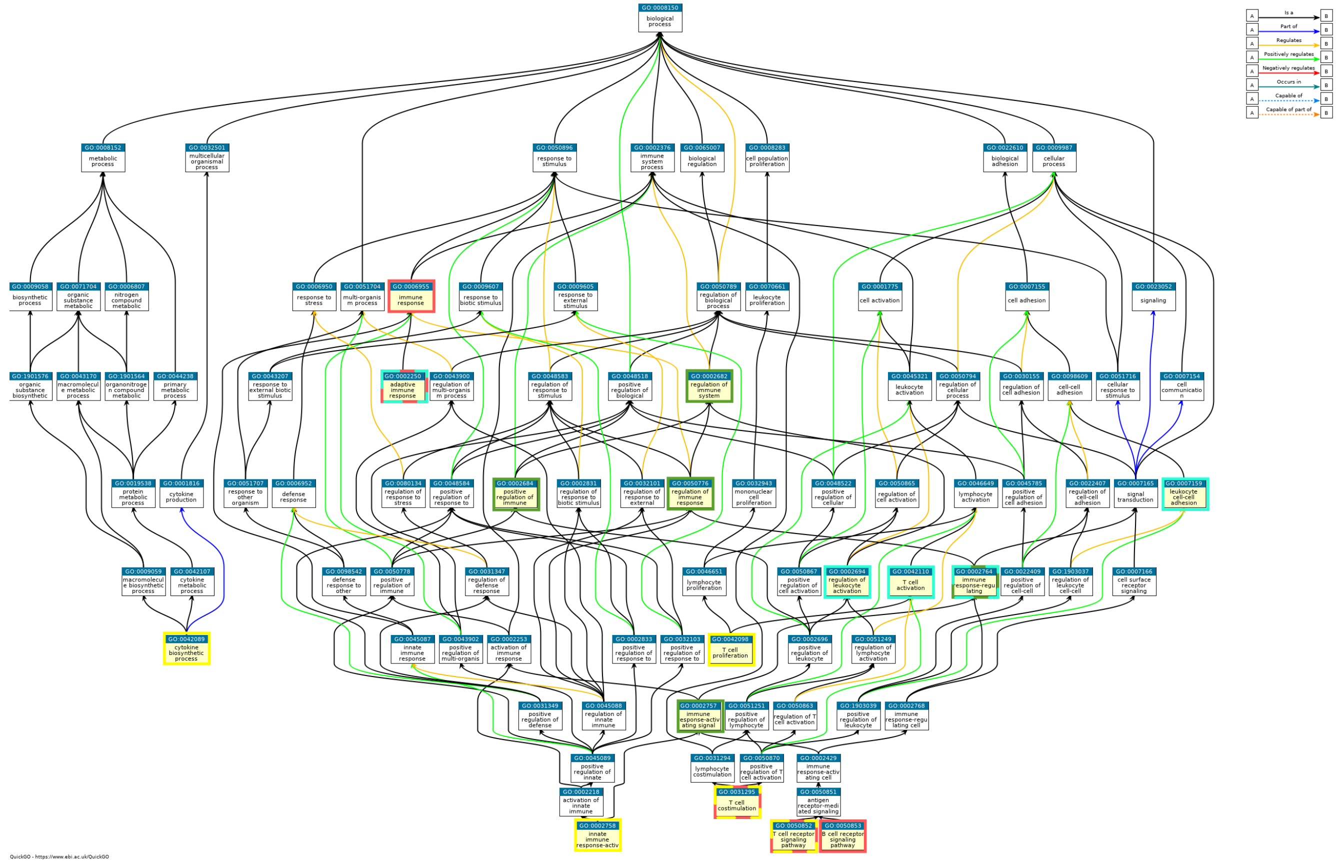

Figure 3: The top 5 terms provided by each evaluated tool positioned along the GO hierarchy represented within an image constructed using QuickGO [14]. The terms provided by WebGestalt are over-framed in blue, in green for g:Profiler, in red for DAVID and in yellow for GSAn.

4 Supplementary details on the quantitative analysis described in the “Comparing GSAn to enrichment tools” subsection

4.1 Description of the investigated gene sets

Figure 4 shows the percentage of genes annotated by GSAn, DAVID [10], g:Profiler [11], ClusterProfiler [12] and WebGestalt [13] for BTM gene sets [9].

A table providing the number of genes in each BTM gene set as well as the number of genes annotated within GOA and by each of the studied tools is available at: https://zenodo.org/record/3662220.

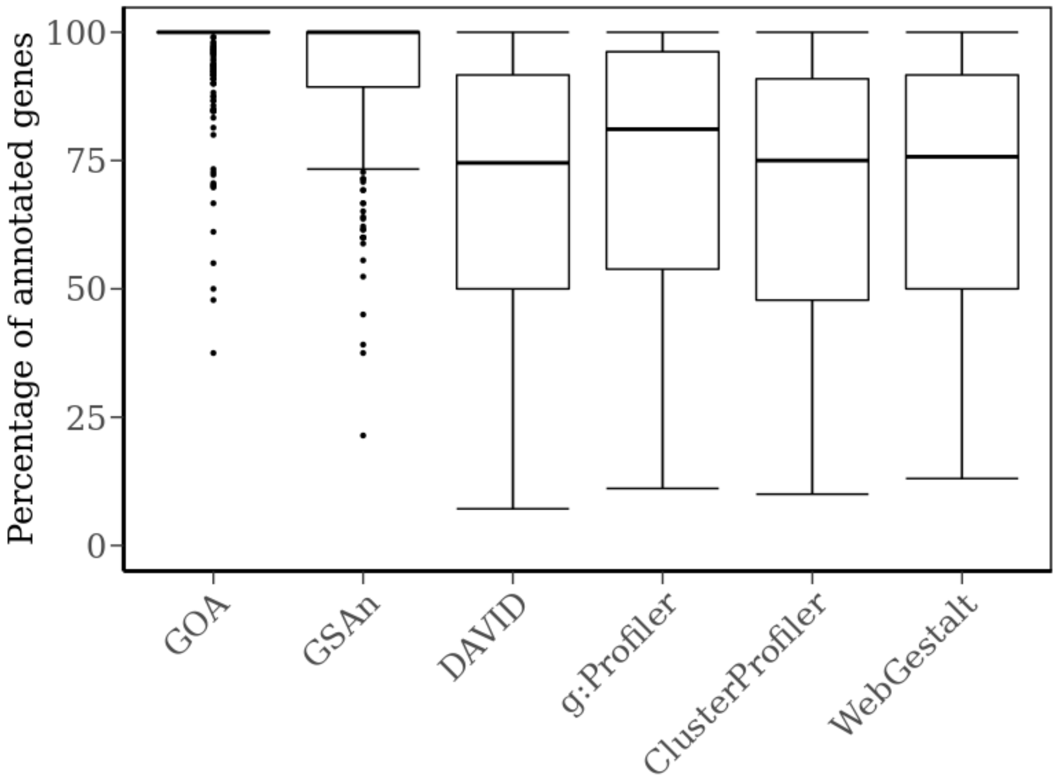

Figure 4: Boxplots displaying the percentage of genes annotated by each tool for BTM gene sets.

4.2 Statistical comparison of results provided by each evaluated tool

To analyze differences between the number of terms and genes according to different IC intervals (from Q0 to Q3), a statistical analysis has been carried out using the unpaired Wilcoxon test with a Bonferroni correction (Figures 5 to 12). We applied a pvalue threshold ($p < 0.05$) for determining statistical significance (colored in red) when results provided by studied tools were different.

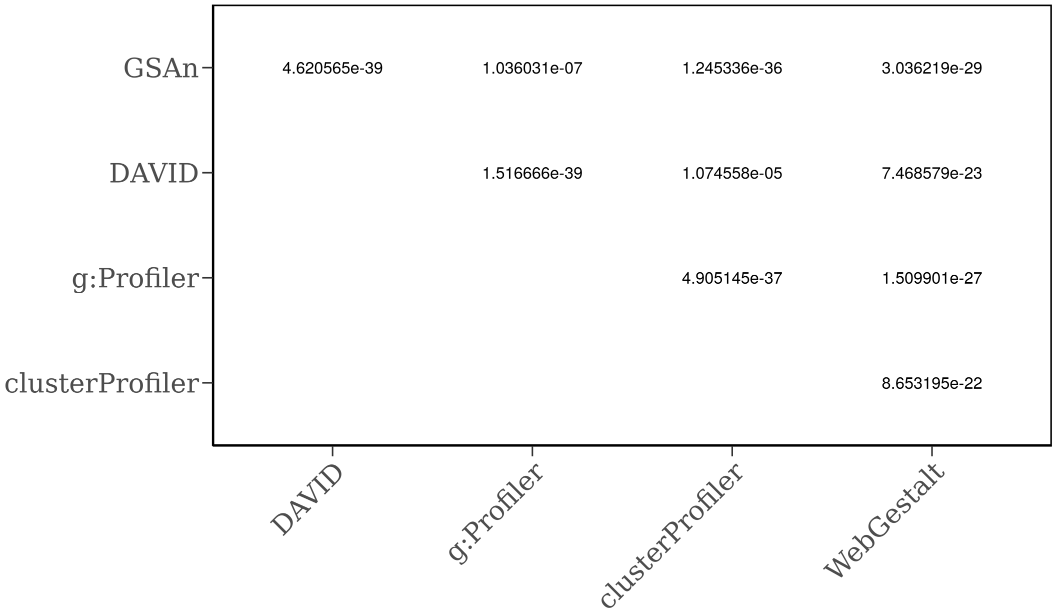

Figure 5: Comparison of the number of terms within the Q0 quartile.

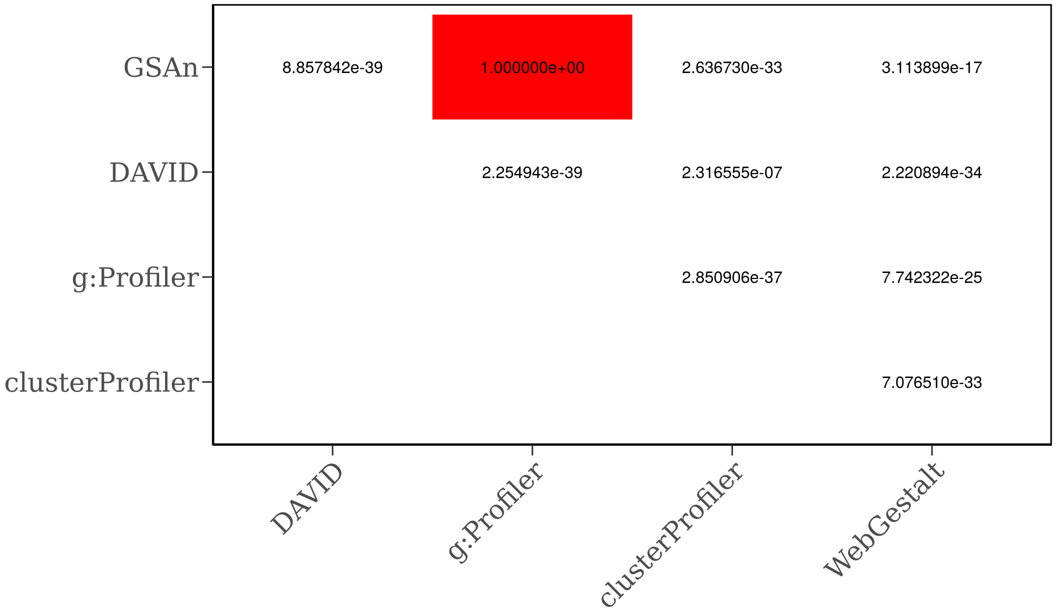

Figure 6: Comparison of the number of terms within the Q1 quartile.

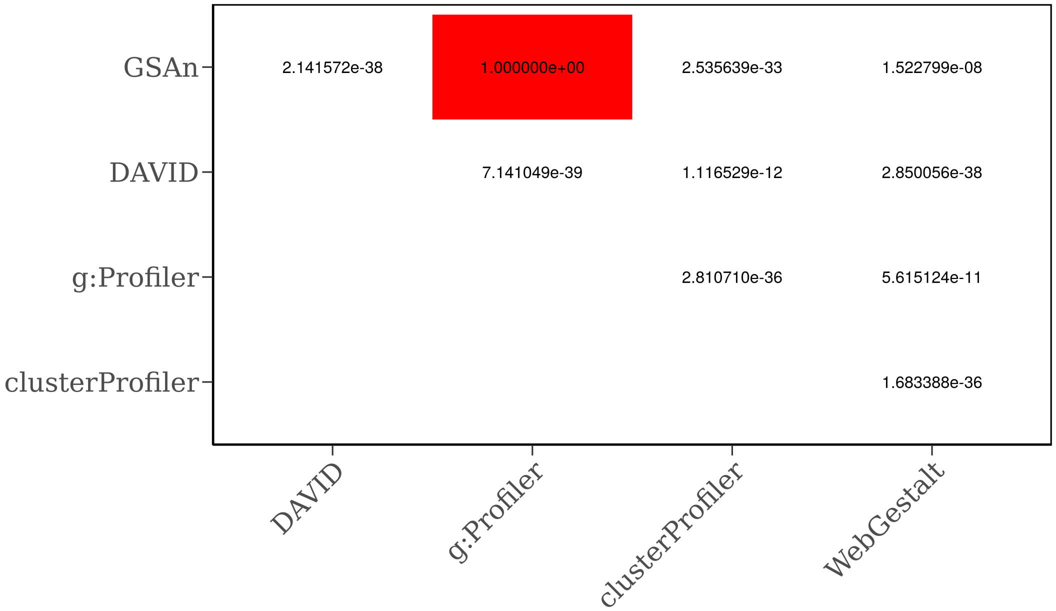

Figure 7: Comparison of the number of terms within the Q2 quartile.

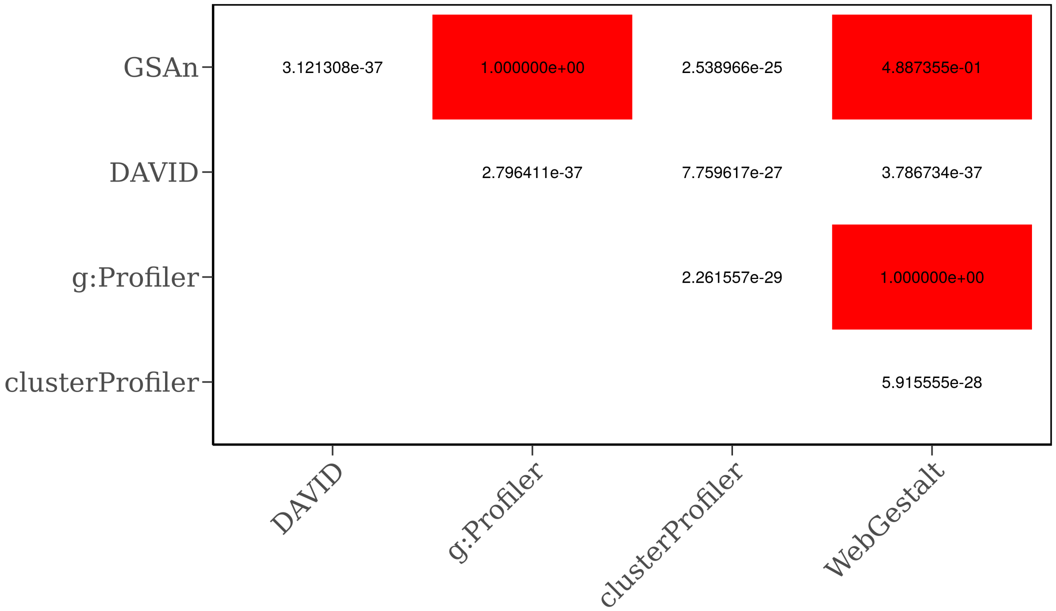

Figure 8: Comparison of the number of terms within the Q3 quartile.

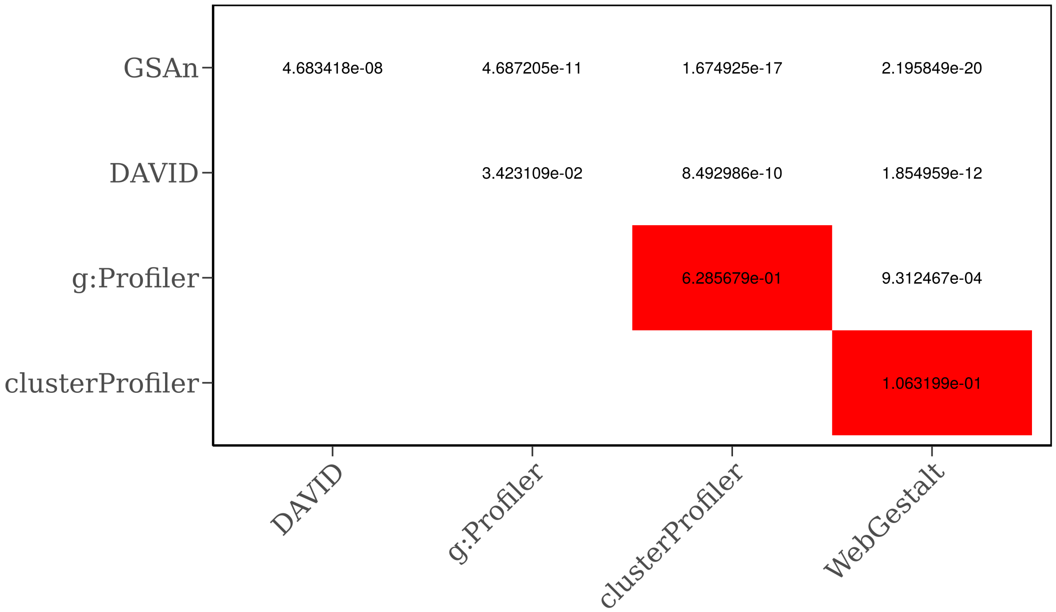

Figure 9: Comparison of the number of genes within the Q0 quartile.

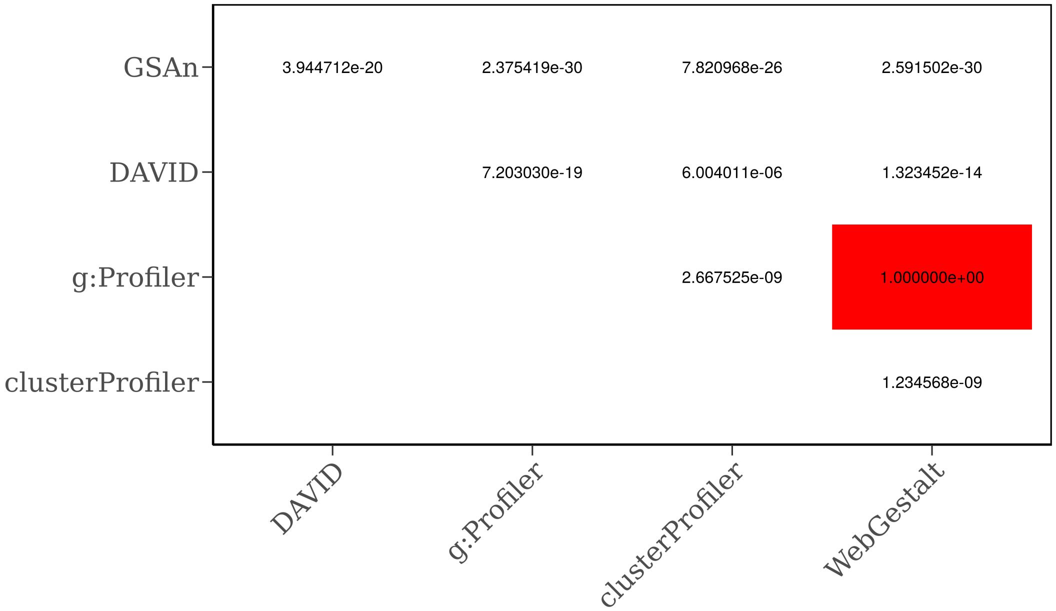

Figure 10: Comparison of the number of genes within the Q1 quartile.

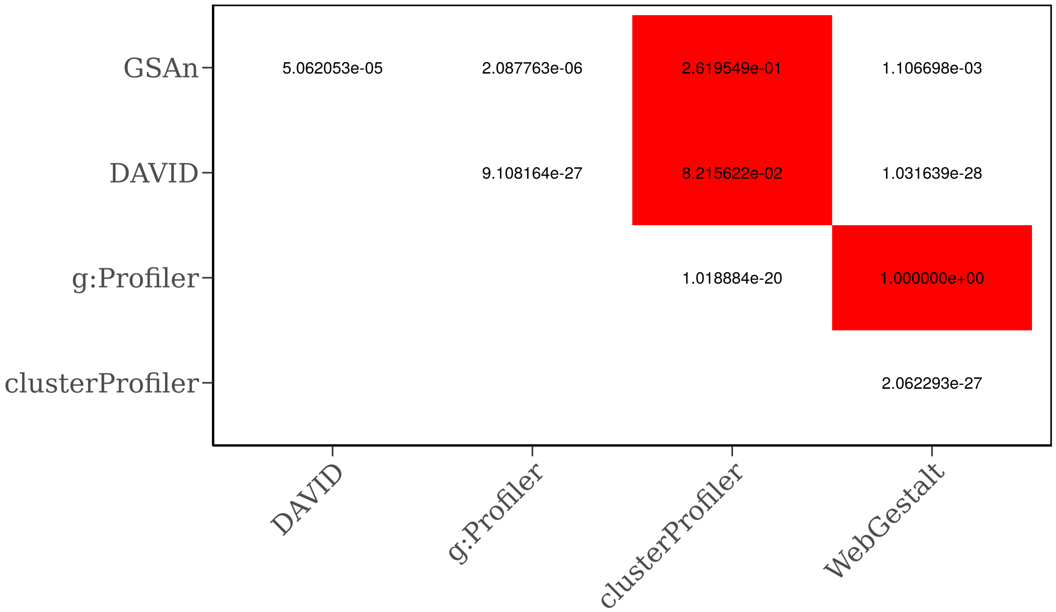

Figure 11: Comparison of the number of genes within the Q2 quartile.

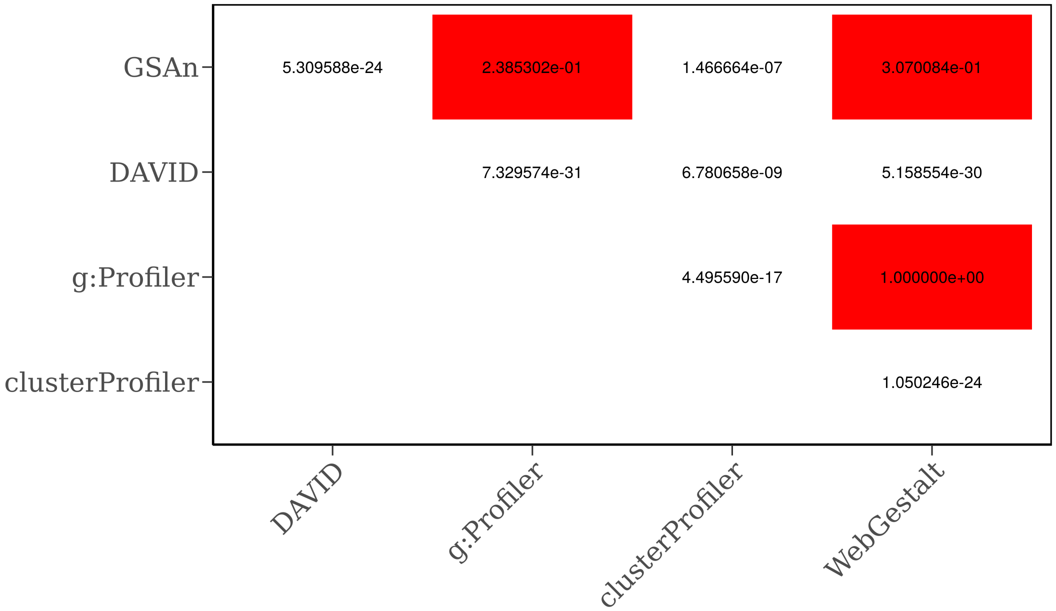

Figure 12: Comparison of the number of genes within the Q3 quartile.

5 Supplementary results by applying GSAn onto real pathway genes

GSAn has been applied onto real pathway genes. Three and four gene sets have been respectively retrieved from Reactome and KEGG databases for homo sapiens and are described in the following section:

Interleukin 7 signaling is composed of 41 genes (see https://reactome.org/PathwayBrowser/#/R-HSA-1266695)

Platelet degranulation is composed of 130 genes (see https://reactome.org/PathwayBrowser/#/R-HSA-114608)

Neutrophil degranulation is composed of 480 genes (see https://reactome.org/PathwayBrowser/#/R-HSA-6798695)

Pyrimidine metabolism is composed of 98 genes (see http://www.gsea-msigdb.org/gsea/msigdb/cards/KEGG_PYRIMIDINE_METABOLISM)

T cell receptor signaling pathway is composed of 109 genes (see http://www.gsea-msigdb.org/gsea/msigdb/cards/KEGG_T_CELL_RECEPTOR_SIGNALING_PATHWAY)

Apoptosis is composed of 87 genes (see http://www.gsea-msigdb.org/gsea/msigdb/cards/KEGG_APOPTOSIS)

Cell cycle is composed of 124 genes (see http://www.gsea-msigdb.org/gsea/msigdb/cards/KEGG_CELL_CYCLE)

Each gene set has then been used to run a GSAn analysis and computed results are available at https://github.com/Ayllonbe/gsan/tree/Release_1.0.1/pathway_tests and can be directly loaded within GSAn web server using the provided json format.

We evaluated the ability of each tool to annotate a set of genes belonging to the same pathway. This type of analysis corresponds to a constrained analysis as the biological function of the studied genes is already known (*i.e.*, these genes are listed in the same gene set because they belong to the same pathway). Ideally, gene set annotation tools should propose the name of the pathway as an annotation with a maximal gene coverage. In this context, we analyzed the different results by identifying, when present, the term(s) (and the number of genes related to this term) that meet the following constraints in order of priority: (1) the term corresponding to the name of the pathway associated with the gene set, (2) the descendant term(s) of the pathway term according to the *is_a* relationship, (3) the descendant term(s) of the pathway term according to the *part_of* relationship, and (4) the descendant term(s) of the pathway term according to the *regulates* relationship.

When analyzing these results (Table 1), it is noticeable that most of the tools propose a term or descendant terms (according to different relationships) that correspond to the name of the pathways, except for *Neutrophil degranulation* and *T cell receptor signaling pathway*. However, even if the analysis highlighted the expected term, some differences can be observed (especially for DAVID) by focusing on the position of the annotation term within the list of terms provided by each tool, the size of this list and the gene coverage. For example, considering

the *Interleukin 7 signaling pathway*, the annotation term *Interleukin 7-mediated signaling pathway* is related to only 2 genes for DAVID, versus 19, 17, 19 and 19 for respectively GSAn, g:Profiler, clusterProfiler and WebGestalt. For other pathways, when the pathway name was not found as such, it is relevant to check if descendant terms have been provided by tools as they give more detailed information. In such cases, it is important to look at the cumulative gene coverage for these sibling terms in order to verify if the gain in information has not been at the expense of the number of genes that are covered by the resulting terms. Three pathways are concerned by this situation (2 for GSAn and 1 for clusterProfiler). Regarding the results for *Pyrimidine metabolism* and *Apoptosis* pathways, GSAn and clusterProfiler propose respectively 5 and 3 descendant terms that annotate 30 and 37 genes. In both cases, a good gene coverage is observed relative to the best gene coverage of other tools. However, for the *Cell cycle*, whereas GSAn proposes more specific terms, the global gene coverage (41 genes) of these 6 terms is smaller than g:Profiler (113 terms). It is noteworthy that this pathway that corresponds to a general term (whose depth is 3 in GO) did not give good results with other tools.

Table 1: Annotation terms of genes being part of the same pathways given by GSAn, DAVID, g:Profiler, clusterProfiler and WebGestalt. The annotation terms corresponding to the name of the pathway are provided or those terms related to this name through an *is_a* relationship (*), a *part_of* relationship (**) or a *regulates* relationship (***).

**Pathways**	**Number of genes**	**Annotation tool**	**Annotation term**	**Position in the term list / Number of terms**	**Gene coverage**
Interleukin 7 signaling	25	GSAn	interleukin-7-mediated signaling pathway (GO:0038111)	2/2	19
		DAVID	interleukin-7-mediated signaling pathway (GO:0038111)	26/80	2
		g:Profiler	interleukin-7-mediated signaling pathway (GO:0038111)	1/8	17
		clusterProfiler	interleukin-7-mediated signaling pathway (GO:0038111)	1/59	19
		WebGestalt	-	0/46	-
Neutrophil degranulation	480	GSAn	neutrophil degranulation (GO:0043312)	1/1	478
		DAVID	-	0/273	-
		g:Profiler	-	0/20	-
		clusterProfiler	***regulation of neutrophil degranulation (GO:0043313)	5/112	4
		WebGestalt	-	0/169	-
Platelet degranulation	128	GSAn	platelet degranulation (GO:0002576)	2/4	121
		DAVID	platelet degranulation (GO:0002576)	1/188	98
		g:Profiler	platelet degranulation (GO:0002576)	1/8	118
		clusterProfiler	platelet degranulation (GO:0002576)	1/88	120
		WebGestalt	platelet degranulation (GO:0002576)	5/122	119
Pyrimidine metabolism	98	GSAn	*UMP biosynthetic process (GO:0006222)	1/13	10
			*pyrimidine deoxyribonucleotide biosynthetic process (GO:0009221)	2/13	5
			*pyrimidine nucleoside salvage (GO:0043097)	3/13	10
			*pyrimidine nucleoside catabolic process (GO:0046135)	4/13	12
			*pyrimidine nucleobase biosynthetic process (GO:0019856)	6/13	13 [30]
		DAVID	pyrimidine nucleotide metabolic process (GO:0006220)	17/122	8
		g:Profiler	pyrimidine nucleotide metabolic process (GO:0006220)	10/11	35

Continued on next page

**Pathways**	**Number of genes**	**Annotation tool**	**Annotation term**	**Position in the term list / Number of terms**	**Gene coverage**
		clusterProfiler	pyrimidine nucleotide metabolic process (GO:0006220)	1/34	34
		WebGestalt	-	0/48	-
T cell receptor signaling pathway	108	GSAn	T cell receptor signaling pathway (GO:0050852)	9/14	40
		DAVID	T cell receptor signaling pathway (GO:0050852)	2/330	39
		g:Profiler	-	0/8	-
		clusterProfiler	-	0/232	-
		WebGestalt	-	0/245	-
Apoptosis	87	GSAn	*neuron apoptotic process (GO:0051402)	13/14	19
		DAVID	apoptotic process (GO:0006915)	3/305	32
		g:Profiler	-	0/12	-
		clusterProfiler	**extrinsic apoptotic signaling pathway via death domain receptors (GO:0008625)	1/189	21
			**extrinsic apoptotic signaling pathway (GO:0097191)	2/189	27
			**execution phase of apoptosis (GO:0097194)	7/189	15 [37]
		WebGestalt	*leukocyte apoptotic process (GO:0071887)	25/194	11
Cell cycle	124	GSAn	**mitotic spindle assembly checkpoint (GO:0007094)	1/16	11
			**DNA damage response, signal transduction by p53 class mediator resulting in cell cycle arrest (GO:0006977)	2/16	19
			**mitotic cell cycle arrest (GO:0071850)	8/16	6
			**meiotic nuclear division (GO:0140013)	12/16	6
			**sister chromatid cohesion (GO:0007062)	13/16	11 [41]
		DAVID	cell cycle (GO:0007049)	46/285	11
		g:Profiler	cell cycle (GO:0007049)	8/11	113
		clusterProfiler	*meiotic cell cycle (GO:0051321)	17/125	26
		WebGestalt	*meiotic cell cycle (GO:0051321)	11/126	26

References

[1] Ayllón-Benítez A., Mougin F., Allali J., Thiébaut R., and Thébault P. A new method for evaluating the impacts of semantic similarity measures on the annotation of gene sets. *PLoS ONE*, 2018;13(11):1–22. doi:10.1371/journal.pone.0208037

[2] Resnik P. Using information content to evaluate semantic similarity in a taxonomy. In *Proceedings of the 14th International Joint Conference on Artificial Intelligence - Volume 1*, IJCAI'95, 1995;pp. 448–453.

[3] Mazandu G.K. and Mulder N.J. in the Gene Ontology. *Advances in Bioinformatics*, 2012;2012:17 pp. doi:10.1155/2012/975783

[4] Song X., Li L., Srimani P.K., Yu P.S., Wang J.Z. Measure the semantic similarity of go terms using aggregate information content. *IEEE/ACM Transactions on Computational Biology and Bioinformatics*, 2014;11(3):468–476. doi:10.1109/TCBB.2013.176

[5] Wang J.Z., Du Z., Payattakool R., Yu P.S., Chen C. A new method to measure the semantic similarity of GO terms. Bioinformatics, 2007; 23(10):1274–1281. doi:10.1093/bioinformatics/btm087

[6] Jain S. and Bader G.D. An improved method for scoring protein-protein interactions using semantic similarity within the Gene Ontology. *BMC Bioinformatics*, 2010;11(1):562. doi:10.1186/1471-2105-11-562

[7] Lin D. An information-theoretic definition of similarity. In *Proceedings of the fifteenth International Conference on Machine Learning*, ICML'98, 1998;pp. 296–304

[8] Quesada-Martínez M., Fernández-Breis J.T., Stevens R., and Mikroyannidi E. Prioritising lexical patterns to increase axiomatisation in biomedical ontologies. *Methods of Information in Medicine*, 2015;54(1):56–64. doi:10.3414/ME13-02-0026

[9] Li S., Rouphael N., Duraisingham S., Romero-Steiner S., Presnell S., Davis C., Schmidt D.S., Johnson S.E., Milton A., Rajam G., *et al.* Molecular signatures of antibody responses derived

from a systems biology study of five human vaccines. *Nature Immunology*, 2013;15:195–204. doi:10.1038/ni.2789

[10] Huang D.W., Sherman B.T., Tan Q., Collins J.R., Alvord W.G., Roayaei J., Stephens R., Baseler M.W., Lane H.C., and Lempicki R.A. The DAVID gene functional classification tool: a novel biological module-centric algorithm to functionally analyze large gene lists. *Genome Biology*, 2007;8(9):R183. doi:10.1186/gb-2007-8-9-r183

[11] Reimand J., Kull M., Peterson H., Hansen J., and Vilo J. g:Profiler—a web-based toolset for functional profiling of gene lists from large-scale experiments. *Nucleic Acids Research*, 2007;35(suppl_2):W193–W200. doi:10.1093/nar/gkm226

[12] Yu G., Wang L., Han Y., and He Q. clusterProfiler: an R package for comparing biological themes among gene clusters. *OMICS*, 2012;16(5):284–287. doi:10.1089/omi.2011.0118

[13] Zhang B., Kirov S., and Snoddy J. WebGestalt: an integrated system for exploring gene sets in various biological contexts. *Nucleic Acids Research*, 2005;33(suppl_2):W741–W748. doi:10.1093/nar/gki475

[14] Binns D, Dimmer E, Huntley R, Barrell D, O'Donovan C, Apweiler R. QuickGO: a web-based tool for Gene Ontology searching. *Bioinformatics*, 2009;15;25(22):3045-6. doi:10.1093/bioinformatics/btp536
